# A Multi-Agent-Based Approach for Semantic Conformance and Interoperability Testing in IoV Environments

**DOI:** 10.3390/s26144550

**Published:** 2026-07-17

**Authors:** Widad Zerzzari, Lalla Amina Charaf, Salma Azzouzi, My El Hassan Charaf

**Affiliations:** Laboratory of Research in Informatics, Faculty of Sciences, Ibn Tofail University, Kenitra 14000, Morocco; lallaamina.charaf@uit.ac.ma (L.A.C.); salma.azzouzi@uit.ac.ma (S.A.); my.charaf@uit.ac.ma (M.E.H.C.)

**Keywords:** multi-agent systems, interoperability testing, internet of vehicles, semantic interoperability, ontology

## Abstract

As part of a successful IoV implementation, testing of IoV-based systems is essential for detecting errors related to device availability, communication reliability, quality of service, and system security. However, this remains a challenge due to the dynamic nature of IoV susceptibility to failures, heterogeneity, and large data streams associated with remotely distributed IoV system components. This paper proposes an intelligent agent-based architecture with a special focus on semantic interoperability testing in order to ensure better interaction and coherence between the various components of the IoV-based system. As a proof of concept, the proposed architecture is implemented in the NetLogo (v6.4.0) multi-agent environment and applied to a case study that detects semantic interoperability issues at both the data and query levels.

## 1. Introduction

The rapid growth of vehicles on the road today leads to increased traffic congestion and an increase in traffic fatalities. As the transportation system evolves, substantial changes are expected, especially in light of the requirements of upcoming vehicles, passengers, and drivers, along with the emergence of technology paradigms such as the Internet of Things (IoT) and cloud computing. Therefore, the development of modern computing and networking technologies has led to the development of a wide range of intelligent devices. Through their interconnection and interoperability, these intelligent devices create a safer and more convenient environment in the transportation context, leading to the revolutionary concept of the Internet of Vehicles (IoV).

In fact, there has been considerable attention given to IoV-based systems, as they allow a safe and efficient exchange of data via wireless communication between vehicles and sensors, vehicles and vehicles, vehicles and roads, and even vehicles and personal devices. A key objective of such systems is therefore to enable coordination between vehicle smart devices, to ensure vehicle privacy protection, and to facilitate collaborative learning in an environment with limited bandwidth and latency requirements.

The successful implementation of these systems nevertheless depends on a number of critical factors, including the availability of devices, communication reliability, service quality, and security. Moreover, the state of both the devices and the overall network may degrade over time, which can affect these factors due to the dynamic nature of IoV, communication constraints, component failures, heterogeneity, and the management of large data streams for remote IoV components. In this regard, testing is a crucial component of an IoV implementation in order to detect any errors that may occur.

Several advanced testing methods are used as part of this process. For instance, conformance tests verify that an implementation meets standards, security tests ensure the safety of connected devices and IoV networks, and semantic tests evaluate the semantic description at the lexical, syntactic, and logical levels. Interoperability tests, in turn, assess whether a device complies with common communication protocols and may be conducted at several levels, including physical, data-type, specification, and semantic levels. However, gathering and analyzing data in IoV-based systems can be difficult, since these systems often interact with users autonomously, run in a highly decentralized environment, and exhibit self-adaptive behaviors.

To address these difficulties, we propose in this paper to test the semantic data interoperability of such systems using the multi-agent system (MAS) paradigm. Agents are software components that can collect information from sensors and environments, perform reasoning, exchange information with other agents, and infer desired goals; they can be proactive, reactive, and able to take decisions autonomously. These properties make multi-agent systems valuable for testing the interoperability of IoV-based systems, as they rely on notions that represent human behavior in terms of thinking, planning, and decision-making. Interoperability between heterogeneous systems further requires a high degree of communication ability in order to understand one another and cooperate toward a common goal. In this context, ontologies can be used to facilitate interoperability by giving meaning to the information exchanged between the different parties, ensuring the correct interpretation of the exchanged knowledge through a common ground vocabulary. The use of MAS in our context is thus required to solve problems that lie beyond the capabilities or knowledge of individual agents: through cooperation between smart devices, agents form a heterogeneous collaborative network and make coordinated decisions.

The novelty of this work lies not in the individual technologies used, but in their integration, which aims to fill an unresolved gap in the literature. Existing approaches to semantic interoperability testing rely on ontologies without taking advantage of agent autonomy and coordination, while multi-agent testing solutions generally lack ontology-based semantic validation. To the best of our knowledge, no previous work combines multi-agent coordination and ontology-driven semantic validation for post hoc, qualitative, “black-box” interoperability testing of IoV data streams equipped with remote monitoring capabilities. Consequently, this work contributes: (i) an integrated architecture combining a multi-agent system (MAS) and an ontology for semantic validation against a reference ontology, (ii) a testing and monitoring strategy adapted to the distributed and heterogeneous nature of IoV environments, and (iii) an ontological formalization of test concepts that promotes extensibility and reasoning.

The remainder of this paper is organized as follows: Section 2 presents a brief description of some preliminary findings. The scope of the study is then explained in Section 3. Section 4 distinguishes semantic conformance testing from interoperability testing. Afterwards, Section 5 describes the design and architecture of the proposed testing prototype. Section 6 reports a case study carried out in the NetLogo environment, and we conclude the paper and outline future work in Section 7.

## 2. Preliminaries

### 2.1. Definitions and Types of Interoperability

There are several types of interoperability that remain relevant in the context of IoV-based systems, as described in [1]:

- Technical interoperability is the ability to build reliable, efficient, and effective collaborative information systems by defining and implementing technical interfaces, standards, and protocols.
- Semantic interoperability is the ability to ensure that the meanings of the exchanged information (i.e., its semantics) are not lost during the interoperability process and can be preserved and understood by users, applications, and institutions, covering messaging formats, structure, and the semantics of the components [2]. This requires effective technical interoperability.
- Organizational interoperability is the ability to identify the actors and organizational procedures involved in delivering a given service and to reach agreement among these actors and procedures on how to structure their interaction. In essence, the system must account for the roles and responsibilities of the entities and actors taking part in the interaction. This requires effective semantic interoperability.

### 2.2. Characteristics of the Interoperability Measurements

As discussed in [3], interoperability is a broad and complex topic, and the development of accurate measures and their application is a difficult task. Nevertheless, assessing interoperability through well-chosen measures is essential to identify the priorities to be considered when networked systems collaborate. We therefore present the existing approaches to interoperability evaluation, characterizing them according to their focus:

- A priori or a posteriori evaluation: The a priori evaluation concerns the model of the system and analyzes its features to verify performance and to allow possible improvement and optimization so that performance requirements are met. The a posteriori evaluation (the test), in contrast, is carried out on the deployed system to measure its performance and to define the actions needed to satisfy its requirements. In this paper, we focus on a posteriori interoperability evaluation (the test) in order to verify whether the expected outputs are reached (validation) during collaborative exchanges in IoV-based systems.
- White-box or black-box evaluation: In the context of testing, studying systems as black boxes consists mainly in examining their inputs and outputs without considering the intrinsic properties that may affect their mutual interactions. Other works argue that studying the semantic interoperability of systems requires deeper insight into the information exchanged, and in particular into its semantics, in order to verify the effectiveness of the exchanges. For this purpose, they suggest opening the systems as white boxes to investigate what happens inside from a semantic point of view.
- Quantitative or qualitative evaluation: Interoperability testing approaches rely on a variety of metrics that may be either qualitative or quantitative. Qualitative metrics are mostly subjective and are built on general evaluation criteria by attaching a maturity level to a specific type of test [4]. Maturity models mostly use qualitative metrics such as pass, fail, or inconclusive. Quantitative metrics define numerical measures to characterize interoperability, such as I-score or DSCC. Some maturity models also define numerical measures, including SoIM, QoIM, MCISI, IAM, and LCIM [5].

### 2.3. IoV: Basic Testing Architecture

There are many difficulties when testing IoV based systems due to their communication constraints, their vulnerability to failures, heterogeneity, and the large amount of data exchanged by remote distributed IoV components.

A number of fundamental concepts underpin the distributed testing architecture of such systems. As part of the testing process, each object within the Internet of Vehicles Implementation Under Test (IoV-IUT),such as vehicles, pedestrian devices, and gateways must be observed through a dedicated interface. The interface defines the control and observation points PCO(s) of the related object. Furthermore, each tester is considered as a component interacting (using the PCO) with an object in the IoV-IUT system.

**Figure 1:**
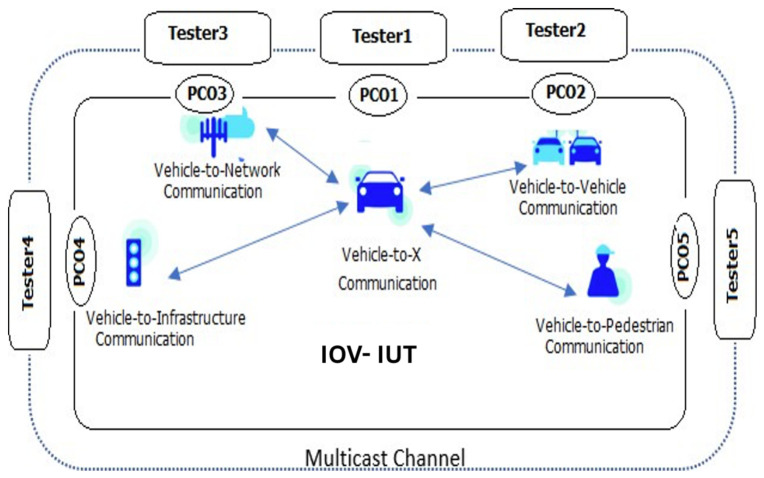
Basic testing architecture.

In this architecture, a PCO is a logical software interface rather than a physical port: it abstracts a single observable interaction of a given IoV object and exposes the messages that can be controlled (sent) and observed (received) at that point. Each PCO is therefore bound to one object and one interaction type, for instance, PCO2 in Figure 1 corresponds to the vehicle-to-vehicle (V2V) interaction observed by Tester2. The binding is one-to-one with the interaction being tested, not with a fixed communication category.

The test architecture is thus composed of a set of testers interacting with the IoV objects via PCO(s) and communicating with each other via a multicast channel as illustrated in the (Figure 1). The multicast channel is the coordination medium shared by the testers. Because each tester observes its object only through its own PCO and has no direct visibility of the other objects, the channel is used to disseminate test stimuli and to exchange the local observations and partial verdicts needed to reach a global verdict on the interaction under test, as well as coordination and synchronization messages if needed. At this level, the channel is an abstract coordination mechanism. Through a series of checks, the method is still a very popular and widely used validation technique when testing implementations of distributed systems. In this case, to stimulate the object, the tester has a given test case and the verdict given by the tester is the result of the interaction with the corresponding object.

## 3. Scope of the Study

### 3.1. Problem Statement

IoV-based systems are networks that link vehicles and other objects as a System of Systems (SoS). Despite all advances in the field, there are still some concerns resulting in potential communication constraints associated with the heterogeneous nature of sensors and vehicles. Therefore, a key challenge is to establish seamless communication between interconnected devices and to address semantic issues in order to ensure a full interoperability between various implementations [6].

Failure to meet the interoperability requirements leads to significant costs, mainly due to the time and resources needed to: (i) process exchanged information and sharing interfaces (technical interoperability) (ii) format data according to a common semantic (semantic interoperability) and to (iii) provide adequate actors needed to adapt the organizational procedures (organizational interoperability). This may have a negative impact on the overall performance as well as the costs and delays to obtain the expected services.

### 3.2. Objectives of the Study

The main objective is to examine how to test and evaluate, a posteriori and qualitatively, whether the requirement for semantic data interoperability between IoV-based systems is met. Therefore, we propose in this paper a comprehensive method to support both the testing and monitoring of semantic data interoperability on IoV-based systems. In fact, information is completely distributed in an IoV ecosystem. This implies that the exchange of information flows is more complex (quantity, diversity, heterogeneity) and may lead to interoperability problems.

For this purpose, the paper outlines a test strategy to evaluate and monitor the semantic interoperability of data transmitted between different objects in IoV systems. Furthermore, given the distribution and diversity of the processes and components involved, the second objective of this study is to present a solution for the implementation of our test and monitoring system using multi-agent systems. The last objective is to formalize the concepts of our system within an ontology in order to push the test model further towards extensibility and interoperability. Thus, it allows verification of the logic consistency of the model (e.g., by using inference engines) and facilitates the sharing of knowledge on the model.

### 3.3. Literature Review

There have been numerous research studies conducted using international standards to provide effective monitoring solutions for interoperability issues.

Accordingly, the authors of [7] describe an approach to semantic interoperability testing in the Internet of Things. They identified three main requirements for semantic tests: (i) lexical checks, (ii) syntactic checks, and (iii) semantic checks. The authors define two types of tests for analyzing semantic interoperability between IoT systems, namely, semantic conformance tests and interoperability tests, along with a detailed test scenario with operational steps. Likewise, a powerful remediation approach is suggested by the authors in [8] to ensure interoperability between locally installed heterogeneous IoT systems. In [9], the authors further reviewed state-of-the-art semantic communications for the IoV, covering the technical background as well as key enabling technologies, including semantic information extraction, communication architectures, and resource allocation and management.

In this case, data mediation and knowledge-based processing are enabled by semantic processing. As part of this study, the authors proposed an architecture for achieving semantic interoperability using international standards, including the oneM2M protocol and OMA NGSI9/10 context interfaces.

However, these standards do not provide full interoperability at the semantic level, whereas they focus on achieving interoperability at the technical (communication) level. In this context, ontologies provide a formal theory about a specific area of discourse and therefore requires a formal formulation in a logical language. To achieve interoperability and semantics between the IoV implementations, several ontologies are used, including: Connected Traffic Data Ontology (CTDO), Vehicle Signal Specification Ontology (VSSo), Security-safety IoV Ontology (SSIoV), Security Assessment Ontology (SecAOnto) [10]. Thus, in order to address data semantic interoperability, a comprehensive ontology for IoT called IoT-O has been developed in [11], which incorporates ontologies representing sensor data, observations, services, quantity types, units, and time. Hence, the authors discuss the extension of the oneM2M standard to support semantic data interoperability based on IoT-O and provide a wide range of benefits of the enhanced standard from real-life scenarios.

The use of ontologies to ensure semantic data interoperability is also discussed in [12,13]. For instance, the authors in [13] present research on applying semantic modeling and ontologies in the IoV sector related to data lifecycle including (i) the ability to collect, reuse, and consolidate data, as well as the role of ontologies in vehicle lifecycle management. (ii) ontologies and semantic interoperability as enabling technologies for the creation of charging location information and traffic information services. Recent research continues to advance semantic technologies for the IoV. For example, ref. [14] surveys this field, identifying core enablers knowledge-based reasoning, learning-driven encoders, context-aware models, and multi modal fusion and demonstrating their value in applications ranging from cooperative perception to hazard detection, where they reduce network load and boost reliability.

Furthermore, the authors in [15] explain what ontologies are and how they can be used in other domains such as computer-aided design, engineering and architectural construction, and geographic information processing in order to facilitate interoperability between software systems. As such, even if these works overcome data semantic interoperability using ontologies (domain concepts, context knowledge, and formal data representation), they lack intelligent ability to understand and interpret exchanged data (organizational interoperability) particularly in a distributed environment.

In this context, artificial intelligence (AI) enables machines to learn from experience, adapt to new data, and perform human-like tasks using rules [16]. With these technologies, computers can be trained to perform specific tasks by processing large amounts of data and recognizing patterns in that data [17,18]. Additionally, some other works [19], compares testing methods using only multi-agent system without ontology support. A discussion of major multi-agent system testing techniques is provided, and the authors conclude by presenting a strategy for conducting testing activities for embedded MAS. More recent work has reinforced the suitability of the multi-agent paradigm for building and testing distributed and IoT-oriented systems, including environments that span connected devices and vehicles [20].

Several studies have also addressed the challenges of distributed testing in large-scale and heterogeneous environments. For example, the authors in [21] propose a testing approach based on temporal data for MapReduce systems. Their work introduces an algorithm for generating local timed test sequences that describe the behavior observed at each test port while reducing the number of messages exchanged among distributed testers. This contribution highlights the importance of coordination mechanisms for efficient distributed testing. Similarly, ref. [22] presents a secure architecture-based conformance testing approach for Infrastructure as a Service (IaaS) cloud environments. Beyond checking conformance with the system specification, the proposed framework verifies compliance with security policies and addresses challenges related to distributed testing through dedicated coordination rules among testers.

However, although these approaches improve the efficiency, coordination, and security aspects of distributed testing, they do not address semantic interoperability issues between heterogeneous systems. In contrast, ontology-based approaches focus on semantic consistency but lack independent coordination mechanisms for distributed testing. This gap motivates the integration of presence-based semantic inference with the distributed multi-agent testing proposed in this work.

The aim of this paper is to propose qualitative metrics to assess the information exchange. The approach is based on the semantic relationships between the systems, considered as black boxes, of an IoV architecture [23]. By taking up these last characteristics, our approach is positioned as described in the (Table 1).

Table 1 contrasts representative semantic interoperability testing approaches along the evaluation characteristics introduced in Section 2.2: the moment of evaluation (a priori versus a posteriori), the visibility of the system under test (white box versus black box), the nature of the metric, and the two enabling technologies of interest here, namely ontologies and software agents. However, several works adopt ontologies for semantic grounding and a smaller number employ agents. Compared to refs. [12,25], our proposal coupling agent-based coordination with ontology-driven validation while performing the evaluation a posteriori, qualitatively, and on black-box implementations. This combination is what differentiates it from other prior semantic-interoperability testing proposals.

## 4. Semantic vs. Interoperability Testing in the IoV Environments

### 4.1. Semantic Conformance Testing

Semantic analysis of data streams from IoV systems can generally be validated in accordance with predefined standards.

**Figure 2:**
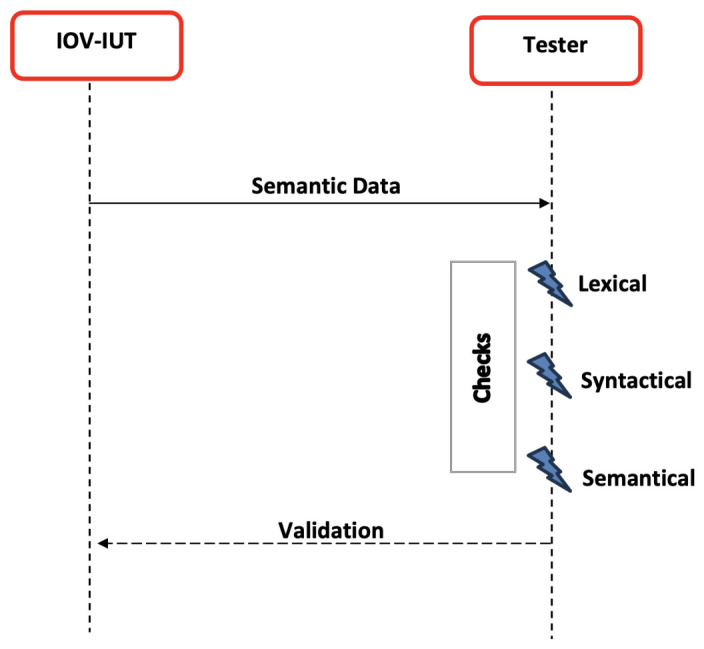
Semantic conformance testing workflow.

As described in the (Figure 2), conformance testing involves interaction between the IoV-IUT and the tester. To ensure high accuracy during the semantic conformance test, the tester checks the semantic data sent by the IoV-IUT and validates the data descriptions at different levels: lexical, syntactic and semantic checks. The final step involves determining whether the semantic data is consistent with the reference ontology [26].

### 4.2. IoV Interoperability Testing

Through interoperability testing, it is determined if a product adhering to a standard specification is interoperable with other products following the same specification. Specifically, the interoperability test here is designed to evaluate whether two implementations can communicate and cooperate effectively.

In other words, interoperability tests involve a series of scenarios designed to evaluate whether a particular implementation can interact with other implementations based on the same specification but implemented by different manufacturers [7]. This is achieved by agreements between requesters and providers regarding, for example, message transmission protocols, procedure names, error codes and argument types. Therefore, they ensure that these exchanges have meaning and that the requester and the provider have a common understanding of the “meanings” of the services and data requested.

Within an IoV architecture, to test data semantic interoperability, we need to verify the interpretation of the data independent of the system under test (IoV-IUT) where the data is being processed. Suppose that there are two systems under test, IoV-IUT1 and IoV-IUT2, which are part of an IoV architecture and communicate with each other.

**Figure 3:**
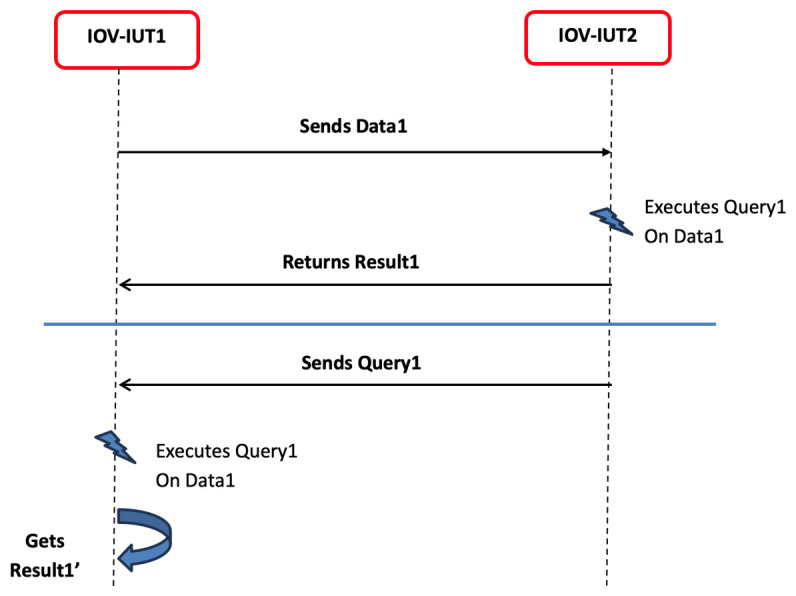
An interoperability testing scenario.

As illustrated in the (Figure 3), IoV-IUT1 and IoV-IUT2 may be considered to be interoperable if the results of semantic processing (the semantic data and the semantic query) are equivalent. In this case, the IoV-IUT1 will compare if Result1 and Result’1 are semantically equivalent as follows:

- The IoV-IUT1 initiates the test by sending the data (Data1) to the IoV-IUT2 that will use such data to run the query (Query1).
- Once the query (Query1) has been executed, the IoV-IUT2 sends both the (Query1) and the execution results (Result1) to the IoV-IUT1.
- Similarly, the IoV-IUT1 will execute the (Query1) using (Data1) and the aforementioned result (Result1’) will then be compared to (Result1).
- If (Result1) is equal to (Result1’), we can deduce that IoV-IUT1 and IoV-IUT2 understand the data in the same way and therefore are interoperable. It should be emphasized that the equality between Result1 and Result1’ is understood here in a semantic, not a purely syntactic, sense: two results are considered equal when they coincide after being resolved against the reference vocabulary, even if their original representations (term IRIs, units, or datatypes) differ. Two structurally different result sets that denote the same information are thus regarded as equivalent, whereas identically structured results carrying divergent meanings are not. The precise comparison procedure canonicalization against the reference ontology followed by multiset equality is formalized in Section 5.5.

## 5. Semantic Interoperability Testing Prototype

Testing IoV-based systems has been the subject of numerous research studies recently in an effort to provide effective monitoring solutions for both semantic and interoperability issues. These studies include the oneM2M protocol and OMA NGSI9/10 context interfaces. The purpose of these standards is therefore not to achieve full interoperability at the semantic level, but rather to achieve interoperability at the level of communication.

Further, the related works section discusses several issues related to semantics and interoperability from the viewpoint of data. In addition, it emphasizes the role of ontologies in the management of vehicle exchanges and how to ensure semantic interoperability between systems by the exchange and sharing of data across diverse tools and platforms. For this purpose, semantic interoperability should ensure that data and information exchanged between the IoV-IUT_i_ implementations are in their intended format and meaning. We describe below our semantic interoperability test prototype:

### 5.1. Overview

**Figure 4:**
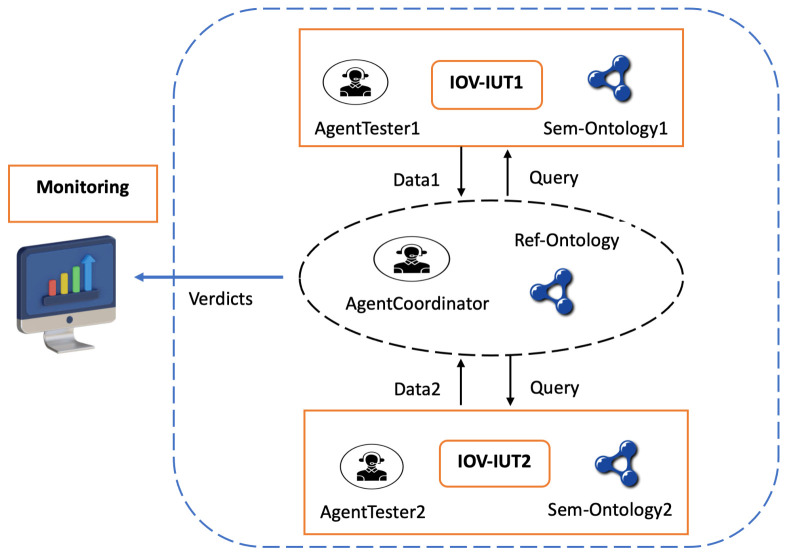
Semantic interoperability testing architecture.

Figure 4 presents the overall architecture of the proposed semantic interoperability testing system, showing the two AgentTesters, each attached to an IoV-IUT and its semantic ontology, the central AgentCoordinator holding the reference ontology, and the monitoring component that collects the verdicts. The role of each element is detailed below.

- IoV-IUT_i_ refers to implementations of the IoV under test. In this regard, these implementations can be thought of as “black boxes”, as their behavior can only be revealed by interacting with their environment or other systems.
- Ontology: An ontology consists of a set of vocabulary and their relationships. In our context, the ontology provides a vocabulary specific to our knowledge domain (IoV) namely Sem-Ontology_i_. As well as being the basis of interoperability, ontologies are used to semantically test the accuracy of IoV data streams in accordance with predefined standards namely: Ref-Ontology. Therefore, each IoV-IUT has its own semantic ontology, while the coordination agent relies on a ref-ontology for semantic interoperability testing. As part of semantic interoperability testing, we examine whether the two IoV-IUTs use the same vocabulary, which confirms semantic interoperability between the two systems at the data level.
- Data: Basically, it is a collection of observations and information gathered from different IoV-IUTs and structured semantically for processing and further analysis.
- Query: The query is intended to produce precise results and to enable the retrieval of derived information based on syntactic, semantic, and structural content.
- AgentTester_i_: used to run semantic tests by collecting data from IUT_i_ and ensuring data transfer to the coordinator Agent.
- AgentCoordinator: is an agent that assumes the role of a coordinator of the agent testers in order to test the semantic interoperability between the IUT_i_.
- Verdicts: The result of each test is expressed using a verdict of three values. A Pass indicates that the implementations agree at the level being tested their vocabularies or query results coincide once resolved against the reference ontology. A Fail indicates a genuine semantic disagreement: the terms or results are well-defined with respect to Ref-Ontology but do not match (for example, one implementation uses velocity while the other uses speed with no alignment axiom relating them). An Inconclusive verdict is reserved for cases where the test cannot be decided at all, because the data falls outside the reference model typically when a term or instance is absent from Ref-Ontology and therefore cannot be canonicalized or compared. The key distinction is that a Fail reflects detected non-interoperability, whereas an Inconclusive reflects the absence of sufficient grounding to reach any verdict. This same logic covers the dynamic and failure-prone nature of IoV environments: if an AgentTester fails to answer the coordinator’s call, or stops responding before returning its data or query results, for example because the underlying vehicle has disconnected the AgentCoordinator has no contribution to canonicalize and compare against Ref-Ontology, and the corresponding round is therefore closed with an Inconclusive verdict rather than a Pass or Fail.

#### Operational Roles of the Agents

To make the behavior of each agent explicit, Table 2 summarizes their inputs, outputs, and decision logic. The AgentTesters act as local probes attached to a single IoV-IUT, whereas the AgentCoordinator is the only entity that holds the reference ontology and therefore the only entity that issues verdicts.

Following the Contract-Net protocol (Section 5.2), the AgentCoordinator acts as the manager that broadcasts the test request, while each AgentTester acts as a contractor that returns the semantic data and query results for evaluation. The coordinator then centralises the comparison so that no individual tester needs knowledge of the other IoV-IUT. This centralized arrangement is the concrete counterpart of the abstract inter-tester coordination introduced in Section 2.3: the multicast exchange of observations is realized here through the AgentCoordinator rather than through direct tester-to-tester messaging.

At setup, the testing environment instantiates one AgentCoordinator together with one AgentTester per IoV-IUT; each AgentTester is bound at creation time to its associated implementation and to its semantic ontology (Sem-Ontology_*i*_), while the AgentCoordinator is the sole holder of Ref-Ontology. Discovery is handled through the Contract-Net interaction itself: when the AgentCoordinator broadcasts its call for proposals, the AgentTesters reachable at that moment reply and thereby register their availability. The set of testers taking part in a given test round is thus determined at the start of the round by which contractors answer the call, rather than being fixed in advance.

### 5.2. Coordination Mechanisms in Our Multi-Agent System

Coordination of agents is crucial for proper interaction in a multi-agent system. In fact, there are a number of reasons why many MAS-based systems should be coordinated:

- A key benefit of agent-based systems is the prevention of chaos. As each agent views the organization from its own perspective, local perspectives, knowledge, and goals may sometimes conflict. Hence, some arrangements must be made to ensure that the individual and collective goals of the agents are achieved.
- For an organization to be considered successful, it must usually satisfy global constraints. There may be instances where a system of agents must work within the constraints of a predefined budget or monitor and report faults of varying priority levels simultaneously on different segments of a network. In order to meet such global constraints, agents must coordinate their behavior.
- The use of distributed expertise, resources, or information in a coherent manner.
- The coordination of agent activities, the sharing of information and resources are essential to solving large and multifaceted problems. The following are some of the mechanisms for coordination in a multi-agent system:
  - **Negotiation**: Agents are involved in an iterative process of exchanging bids as part of the negotiation process.
  - **Auction**: An auction is one of the simplest interaction scenarios. An auction involves a group of agents, with one agent representing the auctioneer and the others representing the bidders.
  - **Argumentation**: The argumentation process is iterative, much like negotiation, but agents can provide reasons for their bids.
  - **Signal broadcasting**: In signal broadcasting, agents send messages to anyone within range. No specific recipient or target is specified.
  - **Contract-Net**: The Contract-Net mechanism is designed for situations where there is a need to negotiate between agents. In the Contract-Net protocol, there are two types of agents: those who initiate negotiations by making requests for proposals, and one or more agents who are to respond to the requests for proposals.

As part of this study, we will enhance the Contract-Net coordination mechanism used for collaborative problem-solving. In this case, the AgentCoordinator will initiate communication with other agents (AgentTesters) so they can address the semantic interoperability issues.

### 5.3. Semantic Validation Based on Ontology

An Ontology is one of the core components to achieve semantic validation. Furthermore, an evaluation of ontologies and annotated data is necessary to determine whether they can be interoperable.

**Figure 5:**
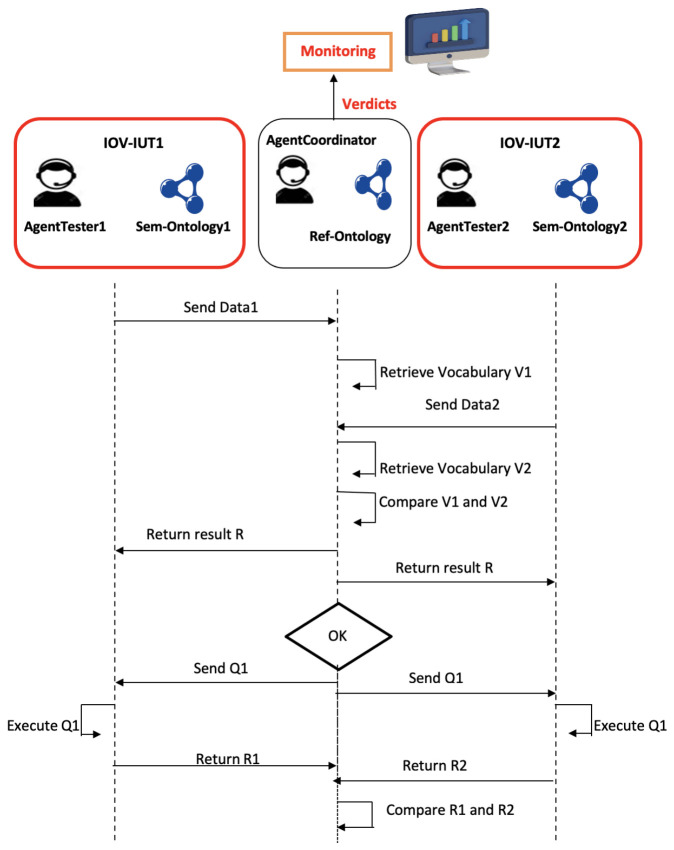
Semantic interoperability testing workflow.

In this perspective, semantic validation testing is introduced to validate the meaning of messages on top of the communication protocol. The validation is carried out using agents that load the ontology and semantically annotated data from the IoV-IUT_i_ and validate them against a reference ontology, as illustrated in (Figure 5). As part of the process, verdicts are sent to monitoring to identify any syntactic or semantic errors.

### 5.4. Semantic Interoperability Testing Process

As described in the previous architecture, this section discusses the principle of semantic interoperability testing. We assume that semantic interoperability refers to the ability to interpret information between various systems, including semantic data and semantic queries. The idea is to check if the final results of semantic processing at the two levels are equivalent.

- A data-level interoperability test: This step examines whether the data provided by the IoV-IUTs share the same vocabulary as defined in the ontology (Ref-Ontology). In order to communicate with AgentCoordinator, AgentTesters should validate the transmitted semantic data (D1 and D2) issued by IoV-IUT1 and IoV-IUT2 for conformance. As illustrated in the figure below, when semantic data (D1 and D2) are received from IoV-IUT1 and IoV-IUT2, the AgentCoordinator retrieves the vocabulary V1 and V2 from both D1 and D2, compares V1 and V2, and returns the results for each IoV-IUT. Therefore, D1 and D2 are interoperable if they have the same vocabulary. We next move on to the query level interoperability test.
- A query-level interoperability test: In a next step, the coordinator sends the same Q1 query to the IoV-IUT1 and IoV-IUT2. Once the implementations have finished executing the query, they send the results back to the AgentCoordinator for comparison and a verdict is then sent to the monitoring station for analysis. As a final conclusion, we will be able to determine whether both implementations can understand the data in a similar manner, thereby enabling their interoperability.

### 5.5. Formal Validation Criteria

To remove ambiguity from the comparison steps described above, we formalize the three checks performed by the AgentCoordinator against the reference ontology Ref-Ontology.

Vocabulary equivalence: Let V1 and V2 be the sets of terms (concept and property IRIs) extracted from the semantic data D1 and D2. Each term t is first resolved against Ref-Ontology through a canonicalization function c(t), which maps t to its reference IRI when an explicit alignment axiom exists (owl:equivalentClass, owl:equivalentProperty, or owl:sameAs), and leaves t unchanged otherwise. The two implementations are vocabulary-equivalent when c(V1) = c(V2) and both are subsets of the vocabulary of Ref-Ontology. A divergent term with no alignment axiom (e.g., velocity where Ref-Ontology defines speed without declaring them equivalent) yields c(V1) ≠ c(V2) and a Fail verdict, whereas a term that does not appear in Ref-Ontology at all (e.g., battery_charge) cannot be canonicalized and yields an Inconclusive verdict.

Query equivalence: Given an identical query Q1 submitted to both implementations, the returned result sets R1 and R2 are first canonicalized: each result tuple is rewritten using the reference IRIs and its literal values are normalized to the units and datatypes declared in Ref-Ontology (e.g., converting km/h and m/s to a single canonical unit). The implementations are query-equivalent when the canonicalized results are equal as multisets, [[R1]]=[[R2]]. Divergence that persists after canonicalization such as a mismatch caused by an unconverted unit on fuel_level produces a Fail verdict.

Semantic consistency: Before any comparison, each annotated dataset is checked for consistency with Ref-Ontology: every instance must be typed by a class of Ref-Ontology, and every property assertion must respect the domain, range, and datatype constraints declared for that property. A dataset that violates a declared constraint is reported as inconsistent and the corresponding case is recorded as Inconclusive, since interoperability cannot be decided on data that does not conform to the reference model. This lightweight check is performed at the assertion level; full OWL DL reasoning over the complete model is left for future work.

## 6. Case Study

To validate the proposed architecture, we implemented a proof-of-concept of the testing system in the NetLogo multi-agent environment. The implementation reproduces the components introduced in Section 5: two AgentTesters, each associated with an IoV-IUT and its semantic ontology (Sem-Ontology_i_), and one AgentCoordinator holding the reference ontology (Ref-Ontology). Coordination follows the Contract-Net mechanism described in Section 5.2, where the AgentCoordinator initiates the exchange and the AgentTesters respond with the semantic data and query results to be evaluated.

### 6.1. Implementation and Experimental Setup

**Figure 6:**
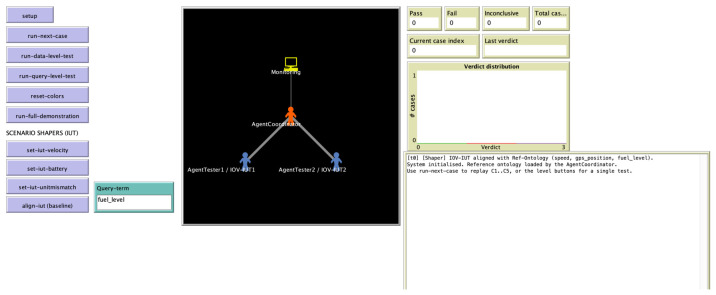
Environment initialization.

We considered a scenario involving two implementations exchanging typical IoV data, namely [speed, GPS position, and fuel level]. The Ref-Ontology defines the shared vocabulary against which each implementation is validated. The verdicts follow the three-valued scheme adopted throughout the paper: Pass, Fail, and Inconclusive (Figure 6).

### 6.2. Results

Table 3 summarizes the verdicts obtained at the data-level and the query-level. At the data-level, when both implementations relied on a vocabulary aligned with the Ref-Ontology, the test returned a [Pass] verdict, confirming that D1 and D2 share the same vocabulary. When one implementation used a divergent term without a corresponding mapping ([e.g., “velocity” instead of “speed”]), the comparison returned [Fail]. A term absent from the reference ontology (e.g., battery_charge) produced an [Inconclusive] verdict, as expected.

At the query-level test, the AgentCoordinator submitted an identical query Q1 to both implementations and compared the canonicalized result sets R1 and R2. When the results matched as multiple sets after unit and IRI normalization, the test returned Pass (case C4). When a divergence persisted because fuel_level was reported in different units without a declared conversion, the canonicalized sets differed and the test returned Fail (case C5).

**Figure 7:**
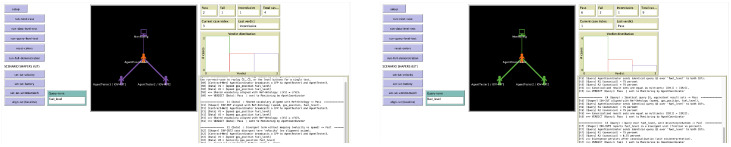
Monitoring dashboard.

Finally, the results show that the proposed multi-agent architecture is able to detect semantic interoperability issues at both the data and query levels and to report them through the monitoring dashboard (Figure 7). While the case study is intentionally limited in scope, it demonstrates the feasibility of the approach and the relevance of the three-valued verdict in distinguishing genuine interoperability from partial or undetermined cases.

## 7. Conclusions

As a summary, testing remains a crucial part of the IoV implementation process to identify potential errors and ensure the smooth deployment of IoV-based solutions. In fact, an efficient testing process will ensure the seamless integration of these solutions. Particularly, a successful verification of semantic interoperability will establish compliance with the standards by examining lexical, syntactic and logical descriptions.

As a proof of concept, we implemented the proposed test prototype in the NetLogo environment and applied it to a case study involving two IoV implementations exchanging semantic data and queries. The results confirmed the ability of the approach to detect data- and query-level interoperability issues and to report them through remote monitoring. Future work will extend the evaluation to larger and more heterogeneous IoV datasets, incorporate a full OWL reasoner for richer semantic validation, and strengthen the runtime robustness of the framework through dynamic agent discovery, re-connection handling, and tolerance to coordinator failure in deployed, non-simulated settings.

## Figures and Tables

**Table 1:** A brief comparison to previous works based on characteristics of the interoperability measurements.

	Interop. Type	Eval. (priori/Posteriori)	Box Type	Metric	Ontology + Agents
[7]	Semantic data	Posteriori	N/R	N/R	×
[8]	Semantic data	A priori	N/R	N/R	×
[24]	Semantic data	A priori	White	Quant./Qual.	×
[12]	Semantic data	Posteriori	N/R	N/R	✓
[13]	Semantic data	N/R	N/R	N/R	×
[25]	Semantic data	A priori	N/R	Quant./Qual.	✓
Our approach	Semantic data/query	Posteriori	Black	Qual. (Pass/Fail./Inconclusive	✓

Note: ✓ = applicable; × = not applicable.

**Table 2:** Roles of the AgentTester and AgentCoordinator agents.

Agent	Input	Output	Decision Logic
AgentTester_*i*_	Semantic data $D_{i}$ and query results $R_{i}$ from its associated IoV-IUT_*i*_; Sem-Ontology_*i*_	Forwards $D_{i}$ and $R_{i}$ (with their vocabulary terms) to the AgentCoordinator in response to a Contract-Net call	None—it does not judge interoperability; it only collects, annotates and transmits
AgentCoordinator	D1, D2, R1, R2 received from the AgentTesters; Ref-Ontology	A three-valued verdict (Pass/Fail/Inconclusive) per test, sent to the monitoring dashboard	Applies the three checks of Section 5.5: vocabulary equivalence c(V1) = c(V2) → data verdict; multiset equality [[R1]]=[[R2]] after canonicalization → query verdict; a term or instance absent from Ref-Ontology → Inconclusive

**Table 3:** Verdicts obtained for the data- and query-level interoperability tests.

Case	Implementations	Test Level	Description	Verdict
C1	IUT1, IUT2	Data	Shared vocabulary aligned with Ref-Ontology (speed, gps_position, fuel_level)	Pass
C2	IUT1, IUT2	Data	Divergent term without mapping (velocity vs speed)	Fail
C3	IUT1, IUT2	Data	Term absent from Ref-Ontology (battery_charge)	Inconclusive
C4	IUT1, IUT2	Query	Identical query Q1; equivalent result sets returned	Pass
C5	IUT1, IUT2	Query	Query over fuel_level; divergent results due to unit misinterpretation	Fail

## Data Availability

The original contributions presented in this study are included in the article. Further inquiries can be directed to the corresponding author.
